# Insulin-Like Growth Factor Binding Protein-3 (IGFBP-3): Unraveling the Role in Mediating IGF-Independent Effects Within the Cell

**DOI:** 10.3389/fcell.2020.00286

**Published:** 2020-05-05

**Authors:** Shailly Varma Shrivastav, Apurva Bhardwaj, Kumar Alok Pathak, Anuraag Shrivastav

**Affiliations:** ^1^VastCon Inc., Winnipeg, MB, Canada; ^2^Department of Biology, University of Winnipeg, Winnipeg, MB, Canada; ^3^Research Institute of Oncology and Hematology, CancerCare Manitoba, Winnipeg, MB, Canada; ^4^Department of Surgery, University of Manitoba, Winnipeg, MB, Canada

**Keywords:** cell survival, apoptosis, IGF-independent role, insulin-like growth factor, nuclear binding partners of IGFBP-3, IGFBP-3 interacting proteins, cell penetration peptides, insulin-like growth factor binding protein-3

## Abstract

Insulin-like growth factor (IGF) binding protein-3 (IGFBP-3), one of the six members of the IGFBP family, is a key protein in the IGF pathway. IGFBP-3 can function in an IGF-dependent as well as in an IGF-independent manner. The IGF-dependent roles of IGFBP-3 include its endocrine role in the delivery of IGFs from the site of synthesis to the target cells that possess IGF receptors and the activation of associated downstream signaling. IGF-independent role of IGFBP-3 include its interactions with the proteins of the extracellular matrix and the proteins of the plasma membrane, its translocation through the plasma membrane into the cytoplasm and into the nucleus. The C-terminal domain of IGFBP-3 has the ability to undergo cell penetration therefore, generating a short 8-22-mer *C*-terminal domain peptides that can be conjugated to drugs or genes for effective intracellular delivery. This has opened doors for biotechnological applications of the molecule in molecular medicine. The aim of this this review is to summarize the complex roles of IGFBP-3 within the cell, including its mechanisms of cellular uptake and its translocation into the nucleus, various molecules with which it is capable of interacting, and its ability to regulate IGF-independent cell growth, survival and apoptosis. This would pave way into understanding the *modus operandi* of IGFBP-3 in regulating IGF-independent processes and its pleiotropic ability to bind with potential partners thus regulating several cellular functions implicated in metabolic diseases, including cancer.

## Introduction

Insulin-like growth factors (IGF), also known as somatomedin C or non-suppressible insulin-like activity, are mitogenic peptides that play an important role in regulating cellular proliferation, growth, differentiation, survival, migration and development. There are two types of IGFs, IGF-I and IGF-II that circulate through the bloodstream from the site of synthesis by liver, which is the primary source. Bound within a heterotrimeric, multi-protein ternary complex along with acid-labile subunit (ALS) and IGF binding proteins (IGFBPs), IGFs are rendered bio-inactive and cannot interact with the receptor (Hodgkinson et al., 1989; Baxter, 1993; Rajaram et al., 1997). Most of the circulating IGFs exists as ternary complex, where the half-life of IGF increases from few min (1–2 min) for free IGFs to more than 12 h (Hodgkinson et al., 1989; Baxter, 1993; Rajaram et al., 1997), however, IGFs can also form binary complex with IGFBPs, where the half-life of IGFs increases to 20–30 min (Baxter, 1993; Rajaram et al., 1997). The release of IGFs from the ternary and binary complex is through the action of proteases (Nakamura et al., 2005), following which the binding of IGFs with type I IGF-receptor (IGF-1R) takes place.

There are six known types of IGFBPs of which IGFBP-3 is the most abundant in the blood circulation. Some proteins like Mac25 also known as IGFBP-7 and proteins that are members of connective tissue growth factor cysteine rich protein (CCN) family possess some structural homology with IGFBPs, however, they lack the affinity for IGFs. Therefore, there is a general consensus that there exists only six IGFBPs as they can bind to IGFs with high affinity, and have a binding constants of 10^9^ L/mol (Baxter, 2000; Forbes et al., 2012).

One of the well-studied roles of IGFBP-3 include the delivery of IGFs to the target cells as its endocrine function. Additionally, the secretion of IGFBP-3 has been reported in different tissues is suggestive of its paracrine or autocrine functions apart from endocrine functions (Cerro et al., 1993; Braulke et al., 1996; Han et al., 1996; Retsch-Bogart et al., 1996; Lindenbergh-Kortleve et al., 1997; Han and Carter, 2000).

Insulin-like growth factor binding protein-3 has been extensively studied in humans, rats and mice (Frystyk et al., 1998; Modric et al., 2001; Muzumdar et al., 2006; Hwang et al., 2008). From an evolutionary perspective, IGFBP-3 is highly conserved in lower organisms. The functions of IGFBP-3 have been classically studied using *in vitro* as well as *in vivo* systems. In drosophila, *in vivo* studies demonstrated that imaginal morphogenesis protein-Late2 (Imp-L2) was capable of binding as well as antagonizing the effects of drosophila insulin-like peptide 2 (Dilp2) (Alic and Partridge, 2008; Honegger et al., 2008) and is a secretory protein. Moreover, overexpression of Imp-L2 could reduce the body size and loss of Imp-L2 increased the body size (Honegger et al., 2008). These studies strongly support that Imp-L2 plays a role similar to IGFBPs. Transgenic mice overexpressing IGFBP-3 demonstrated a similar phenotype (Nguyen et al., 2015). In a study led by Nyomba, transgenic mice overexpressing human IGFBP-3 under the control of phosphoglycero kinase promoter demonstrated a reduction in body weight compared to their wild type littermates (Nguyen et al., 2015). On the contrary, IGFBP-3 knockout mice did not show significant difference in body weight in comparison with the wild type littermates (Scully et al., 2016).

### The Structure of IGFBP-3

**FIGURE 1:**
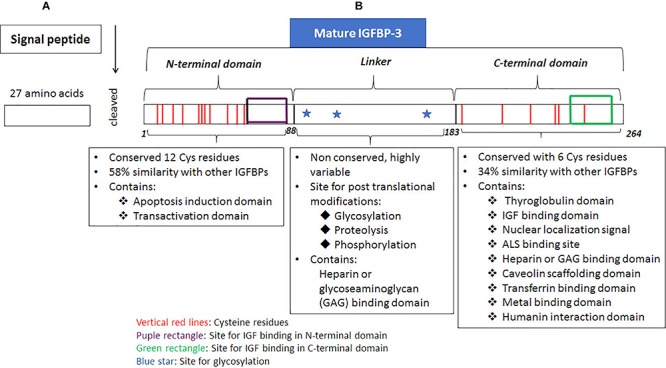
Structure of IGFBP-3. Translation is initiated on the ribosomes, **(A)** signal sequence, a 27 amino acid region present on the *N*-terminal end of the nascent IGFBP-3 polypeptide chain is responsible for targetting it onto the surface of rough endoplasmic, which is recognized by the signal recognition particle (SRP). Through the concerted action of two G-proteins, namely SRP that binds to the signal sequence; and SRP receptor that is present on the endoplasmic reticulum (ER), the nascent polypeptide chain with the signal sequence binds onto a channel protein, translocon along with the ribosome, mRNA. The nascent poplypeptide chain of IGFBP-3 is translated on ER and through the translocon moves into the lumen of ER where the signal sqence is excised by proteases. **(B)** Mature IGFBP-3 consists of *N*-terminal domain, linker domain and the *C*-terminal domain. Posttranslational modification of glycosylation occurs in the lumen of ER and Golgi complex in the mid-linker domain of IGFBP-3. Various domains of IGFBP-3 protein and their associated functions are dipicted in the figure.

All the precursors of IGFBPs possess secretory signal peptide sequence (Bhattacharyya et al., 2006), including IGFBP-3, which possesses a 27 amino acid, signal peptide at the *N*-terminus. The signal peptide is excised when the nascent polypeptide chain of IGFBP-3 is transferred into the endoplasmic reticulum (Figure 1). Mature human IGFBP-3 comprises of 264 amino acids and possesses three structural domains including NH_2_-terminal (*N*-terminal) domain, central or linker region and COOH-terminal (*C*-terminal) domain (Figure 1). Of these the linker region is non-conserved, whereas the *N*-terminus and the *C*-terminus are both cysteine-rich and highly conserved domains. *N*-terminal domain has 12 cysteine residues of which intradomain disulphide bridges are formed between the residues 13–40, 16–42, 24–43, 31–46, 54–61 and 67–87; the *C*-terminal domain has 6 cysteine residues of which 186–213, 224–235, and 237–258 forms the intradomain disulphide bridges (Jafari et al., 2018). A mature deglycosylated form of human IGFBP-3 has a molecular mass of about 28.7 kDa based on the amino acid sequence, however, the molecular weight can range between 46–53 kDa due to glycosylation of IGFBP-3 that can occur at three positions. The *N*-glycosylation sites have a consensus sequence of Asn-X-Ser/Thr on IGFBP-3 (Firth and Baxter, 1999). The three glycosylation sites include Asn89, Asn109 and Asn172 (Figure.1; Firth and Baxter, 1995). Glycosylation at Asn89 and Asn109 contributes to ∼4 kDa and 4.5 kDa of carbohydrates, respectively, whereas, Asn172 is either not glycosylated or contributes to 5 kDa due to glycosylation, thus resulting in the formation of characteristic doublets of IGFBP-3 that can be detected in Western blot analyses (Firth and Baxter, 1995). The *N*-glycosylation of IGFBP-3 has been shown to alter the binding with glycoseaminoglycans (GAG), which are present on the cell surface. Other posttranslational modification includes phosphorylation, which have been demonstrated to occur at Ser111, Ser113 and Ser156 residues are catalyzed by casein kinase 2 (Cobb et al., 2009) and Ser129, Ser174, and Ser156 residues are catalyzed by DNA-dependent protein kinases (DNA-PK). The *N*-terminal domain of IGFBP-3 extends between amino acid residues 1–87 the central linker domain contains 95 amino acids ranging between 88–183 whereas, the *C*-terminal domain extends between 184–264, (Yamanaka et al., 1999). IGF binding domains of IGFBP-3 can be found in both the *N*-terminus as well as the *C*-terminus (Yan et al., 2004). The *N*-terminal domain of IGFBP-3 is required for inducing apoptosis (Shahjee et al., 2008) and binding with transcription factors (transactivation domain) (Zhao et al., 2006; Zhong and Duan, 2016). The central linker domain of IGFBP-3 is not known to possess the IGF binding capabilities rather it is the site for posttranslational modification like glycosylation, phosphorylation and proteolysis apart from this it also possesses a GAG or heparin binding domain. The posttranslational modifications can alter the 3D-confomation of the molecule altering the cell interactions, the phosphorylation and catalysis by proteases, which in turn can alter both the IGF-dependent as well as IGF-independent processes regulated by IGFBP-3 (Firth and Baxter, 2002). The secondary structure of IGFBP-3 is similar to thyroglobulin-type 1 molecule and in the proper 3D-conformation state, the *C*-terminal domain of IGFBP-3 resembles thyroglobulin (Payet et al., 2003; Jafari et al., 2018). The thyroglobulin type 1 domain extends between residues 183–258 (Jafari et al., 2018), *vide infra*, Figure 1. IGFBP-3 possesses a bipartite NLS in the *C*-terminal domain, which includes a stretch of two clusters of basic amino acids separated by a spacer region (Bhattacharyya et al., 2006). Apart from NLS, *C*-terminal domain also includes, transferrin-binding region, caveolin scaffolding domain (Lee et al., 2004), heparin binding domain or GAG binding domain (Smith et al., 1994; Fowlkes and Serra, 1996; Liu et al., 2014), metal binding domain (Singh et al., 2004), and ALS binding domain (Firth et al., 1998b), *vide supra* Figure 1. Therefore, the *C*-terminal domain of IGFBP-3 plays an important role in metal binding, cellular uptake, as well as nuclear localization of the protein.

### IGF-Dependent and Independent Roles of IGFBP-3

Insulin-like growth factor (IGF) binding protein-3 can either trigger the activation of IGF-dependent signaling, which are a part of IGF-dependent roles of IGFBP-3 or can perform IGF-independent actions.

#### IGF-Dependent Role of IGFBP-3

Insulin-like growth factor-dependent roles of IGFBP-3 include the facilitated delivery of IGFs to its cell surface receptors and activation of its associated downstream signaling cascade. IGFs mediate their action through their binding with IGF-1R, which is a transmembrane protein belonging to the family of receptor tyrosine kinases (RTK). Both IGF-I and IGF-II can bind with IGF-IR to mediate their effects, however, the binding of IGF-II with IGF-1R is lower in affinity. IGF-II can also bind with type 2-IGF receptor (IGF-2R) (Nolan et al., 1990). While IGF-1R is a hetero-tetramer comprising of two alpha subunits and two beta subunits (Chitnis et al., 2008), on the contrary, IGF-2R is monomeric and does not initiate downstream signaling (Braulke, 1999). IGF-2R also functions as a mannose-6 phosphate receptor that binds with the lysosomal enzymes possessing characteristic mannose-6 phosphate residues. The binding of IGFs to the IGF-1R triggers the activation of its intrinsic tyrosine kinase activity that results in trans-auto-phosphorylation of the receptor (Girnita et al., 2014). The activated receptor in turn activates the downstream signaling through phosphatidylinositol 3-kinase (PI 3-kinase)/ protein kinase B (PKB)/ mammalian target of rapamycin (mTOR) pathway (Latres et al., 2005) and mitogen activated protein kinase pathway (MAP kinase) (Peruzzi et al., 1999).

#### IGF-Independent Role of IGFBP-3

**FIGURE 2:**
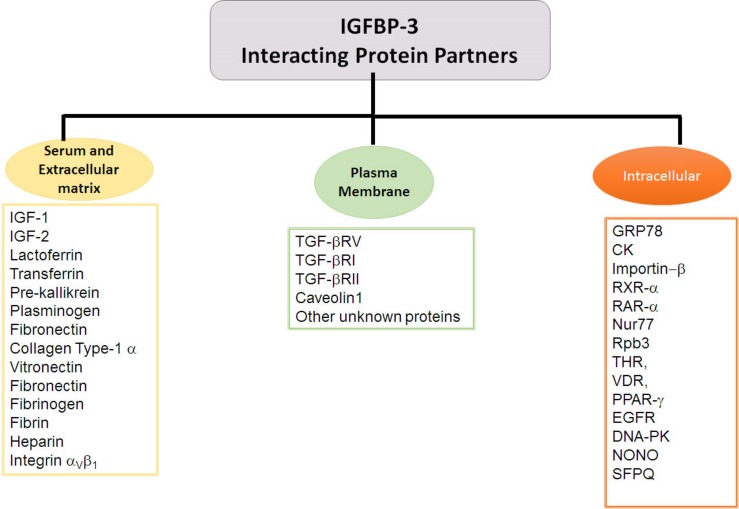
IGFBP-3 binding partners. Flowchart exhibiting IGFBP-3 interacting proteins partners delineated based on the extracellular and cellular localization.

Insulin-like growth factor-independent roles of IGFBP-3 include its association with the extracellular matrix, its association with the proteins on the plasma membrane, its translocation across the plasma membrane into the cytosol and its nuclear localization. This is achieved through the interactions of IGFBP-3 with several proteins, *vide infra* for the list of various proteins that can interact with IGFBP-3 (Figure 2).

## Igfbp-3 Interactions With Proteins of Serum and Extracellular Matrix (ECM)

Either upon the delivery of IGFBP-3 from its site of synthesis through the bloodstream bound within a ternary complex, as an endocrine function or upon its secretion by the cell where it can mediate its autocrine or paracrine actions, IGFBP-3 independent of IGFs can translocate from the extracellular matrix (ECM) into the cytoplasm. IGFBP-3 is known to interact with several proteins in the serum including lactoferrin (Baumrucker, 2000) and transferrin (Weinzimer et al., 2001). IGFBP-3 binding affinity with iron-saturated transferrin is twice in comparison with apo-transferrin alone (Weinzimer et al., 2001). While the binding with lactoferrin and transferrin competes with IGF binding, IGFBP-3 interactions with pre-kallikrein, plasminogen, fibrinogen, (Campbell et al., 1999) and fibronectin (Gui and Murphy, 2001) are not impacted by IGF ligand occupancy, suggestive of the fact that these are IGF-independent interactions of IGFBP-3. Apart from these, IGFBP-3 can interact with the other proteins of ECM like type-I α collagen (Liu et al., 2003), vitronectin (Kricker et al., 2010; Kashyap et al., 2016), fibrinogen, fibrin (Campbell et al., 1999), and heparin (Martin et al., 1992; Fowlkes and Serra, 1996; Durham et al., 1999).

## Cellular Uptake of Igfbp-3

### Metal Binding Ability of IGFBP-3 and Its Role in Cellular Uptake

Insulin-like growth factor binding protein-3 has been demonstrated to possess metal binding capabilities (Singh et al., 2004; Huq et al., 2009; Miljus et al., 2013). Singh et al. (2004) have demonstrated that a 12-mer peptide of IGFBP-3 containing loop rich in cysteine possesses a Zn^+2^ finger-like motif and can bind with nitrilotriacetic acid (NTA)-immobilized metal affinity columns, therefore this region is referred to as the metal binding domain (MBD). Full length IGFBP-3 has been demonstrated to bind NTA column charged with metal ions, including nickel, cobalt, iron, zinc, magnesium, and manganese but not calcium (Singh et al., 2004). The study demonstrated that Ni- NTA and ferrous-NTA binding with IGFBP-3 were inhibited by IGFs, indicative of its IGF-dependent effect. MBD has been proposed to play a role in the nuclear uptake of IGFBP-3. Although, the metal binding to IGFBP-3 is an IGF-dependent process yet its physiological response was an IGF-independent process. A 14-mer peptide containing MBD could induce apoptosis in stressed HEK293 cells similar to IGFBP-3 in an IGF-independent manner (Singh et al., 2004). IGFBP-3’s role in the mediation of apoptosis includes its actions within the nucleus. MBD peptide that included the NLS and putative caveolin binding domain caused cellular and nuclear uptake of proteins fused with GFP and streptavidin-HRP (Singh et al., 2004). In fact MBD region of IGFBP-3 has been reported to interact with integrin α_*v*_ and β_1_, caveolin-1 and transferrin receptor suggestive of its role in associations with the cytoskeletal system (integrin), iron transport (transferrin) and membrane lipids (caveolin) of cells. Antibodies against integrin and transferrin could reduce the cellular uptake of unrelated chimeric proteins tagged with GFP or streptavidin-HRP (Singh et al., 2004).

### GAG Domain of IGFBP-3 Required for Cell Penetration Peptide (CPP) Properties

Cell penetration peptides or peptide transduction domains (PTD) are class of peptides that are known to traverse the plasma membrane (Madani et al., 2011). Positively charged i.e., polycationic molecules CPPs or PTDs are known to deliver molecules within mammalian cells. Polycationic molecules initially associate with the cell surface heparan sulfate or GAG, through their heparin or GAG binding domain involving electrostatic forces of interactions followed by internalization through endocytic pathways, either enclosed within vesicles (endocytosis) or without vesicles (direct transport) (Madani et al., 2011). Cells are known to internalize heparan sulfate proteoglycans through endocytosis and along with this they could potentially internalize external ligands that associate with their GAG domains (Fuchs and Raines, 2004; Goda et al., 2008; Liu et al., 2014).

Mechanism through which IGFBP-3 interacts with the plasma membrane of the cells or ECM include its ability to associate with GAG through the C-terminal domain (Singh et al., 2004; Liu et al., 2014). The amino acid region spanning from 215 to 232 residues in the *C*-terminal domain was characterized as a GAG-binding domain, which is important for the internalization of IGFBP-3 (Singh et al., 2004). Proteoglycans are glycoproteins that possess one or more of GAG. Heparin is a highly sulphated glycoprotein. IGFBP-3 can associate with GAGs, heparin, and heparan sulfate through its GAG binding domain.

Matrix metalloprotease (MMP)3 and MMP9 can bind with IGFBP-3, of these, MMP3 has been demonstrated to cause the proteolysis of IGFBP-3 into six fragments four of which retained the ability to bind with heparin-agarose. Fowlkes et al. demonstrated that IGFBP-3 contains at least two heparin-binding domains, one in the mid-linker region and another in the conserved, *C*-terminal domain of IGFBP-3 (Fowlkes and Serra, 1996) Figure 1. Heparin or GAG binding domain, NLS and transferrin binding domains overlap each other. Heparin binding potentially leads to the association of IGFBP-3 with the plasma membrane of cells. Several studies have demonstrated that the addition of heparin reduces IGFBP-3 association with fibroblast cell monolayers (Yang et al., 1996) and sertoli cells from rat (Smith et al., 1994). Association with heparan sulfate or GAG is important in bringing IGFBP-3-IGF complex in close vicinity with IGF receptor and in mediating its IGF-dependent functions of IGFBP-3. Localization of IGFBP-3 is important for the potentiation of IGF action and the association of IGFBP-3 with the cell surface receptors or ECM reduces the affinity of IGFBP-3 for IGFs thus making them available for their receptors (McCusker et al., 1990; Conover, 1992).

Hiroaki et al. reported of an 18-mer peptide from IGFBP-3 containing heparin binding domain that resulted in the cellular uptake of IGFBP-3 as well as unrelated proteins, moreover, competition with heparin could inhibit this activity of the 18-mer peptide (Goda et al., 2008). The study proposed the possibility of both direct as well as caveolae-mediated endocytosis as competitive inhibition with heparin is common to both the pathways of internalization. The same study also reported that the protein delivery efficiency of this 18-mer peptide of IGFBP-3 was 80–150% of that of HIV-TAT, a known potent protein transduction domain (PTD) (Goda et al., 2008).

Other researchers demonstrated that the cellular uptake of IGFBP-3 and unrelated proteins tagged with GAG binding domain of IGFBP-3 could be mapped to a 14-mer peptide containing residues 223–236 (Singh et al., 2004). The cellular uptake property of IGFBP-3 could be further reduced to an even shorter 12-mer peptide, a Cys-Cys loop containing residues 224–235 (Singh et al., 2004), which is beyond the putative heparin-binding domain.

A recent study demonstrated that the cellular uptake function of GAG binding domain and the physical GAG-binding functions of IGFBP-3 can be attributed to the distinct regions within the *C*-terminal domain (Liu et al., 2014). Mutants of GAGs possessing defects in truncation of GAG could affect the cellular uptake of IGFBP-3 ranging from impaired cellular uptake to complete inability to internalize (Liu et al., 2014). In the same study, Liu et al. (2014) demonstrated that IGFBP-3 peptides entered as a CPP in a GAG-dependent manner. An 18-mer peptide including the GAG domain of the *C*-terminus was synthesized and of these, 8-mer peptides retained a stronger binding to GAGs, whereas 10-mer peptides retained most of the cellular uptake functions.

#### Biomedical Applications of Short Peptides From the *C*-terminal Domain of IGFBP-3 as a CPP

Therapeutic drugs or genes have to be internalized into the cells to carry out their therapeutic actions or for diagnostic purposes, thus have to cross the plasma membrane. CPPs can enter the cells irrespective of the presence of receptor on the membrane and tend to exhibit low toxicity, however, they lack specificity and are unstable. CPPs have the ability to assist in carrying cargo moieties without causing injuries to the cell, thereby augmenting the bioavailability of the drug hence improved therapeutic efficiency. For a detailed review on cell penetration peptides please refer to (Habault and Poyet, 2019; Shafiee et al., 2019).

8–22 mer peptides from the *C*-terminal domain containing GAG domain and the NLS sequence could successfully transport unrelated proteins tagged to them within cells ([Bibr B146][Bibr B56]; Liu et al., 2014).

Huq et al. investigated the possibility of improving chemotherapy through the use of biological modifier peptides. They demonstrated that 22-mer peptides of IGFBP-3 from the *C*-terminal domain containing the MBD that functions as a transporter domain could also selectively target the cancer cells (Huq et al., 2009). The study determined the efficacy of these peptides using *in vitro* cell cultures as well as in *in vivo* mice and rat models (Huq et al., 2009). The MBD tagged proteins and peptides were reported to be localized in kidneys and pancreas. *In vitro* studies determined that the cellular uptake of these peptides correlated with the expression of stress genes, which was confirmed using microarray (Huq et al., 2009). The peptides were marginally toxic, similar to 5-fluorouracil. Upon the injection of CCRF-CEM T-cell leukemia or MDAMB-435 breast cancer cells into Rag-2 mice followed by administering a cocktail of MBD-derived peptides of IGFBP-3 from the *C*-terminal domain a reduction in the burden of cancer within 7 days was observed as demonstrated by reduced splenomegaly, and reduced bone marrow cancer cell burden (Huq et al., 2009). Therefore, short peptides from the terminal domain of IGFBP-3 possessing CPP could prove to be beneficial in designing CPP-assisted drug or gene-based therapeutics. One of the major advantage for IGFBP-3 CPP over others, as demonstrated experimentally, is the ability to specifically target the cancer cells (Huq et al., 2009). Low toxicity, specificity of targeting cancer cells for therapy using CPPs from the C-terminal domain of IGFBP-3 could potentially change the treatment regimen for cancer in the future and more research is required in this area.

### Mechanisms of Cellular Uptake of IGFBP-3

The transport of IGFBP-3 across the plasma membrane involving different mechanisms of endocytosis has been demonstrated, including the classical clathrin-dependent endocytosis, and through caveolae or lipid rafts and fluid-phase uptake.

#### Classical Cellular Uptake of IGFBP-3 Through Clathrin-Coated Pits

Insulin-like growth factor binding protein-3 can interact with transferrin (Weinzimer et al., 2001) and it has been proposed that transferrin in turn can interact with its receptor, thus enabling the internalization of IGFBP-3 through receptor-mediated endocytosis along with transferrin (Lee et al., 2004). Transferrin receptor is considered as the canonical clathrin coated pit marker. When transferrin receptor was inhibited using a transferrin receptor antibody in cells treated exogenously with IGFBP-3, a decline in the accumulation of IGFBP-3 in the nucleus was reported (Lee et al., 2004). It was further demonstrated that TNF-α under serum-deprived conditions resulted in increased synthesis of IGFBP-3, which is secreted and internalized into the cells. When cells were treated with transferrin receptor antibody along with TNF-α there was a decrease in intracellular as well as nuclear IGFBP-3. Thus, supporting the role of transferrin-receptor mediated internalization of IGFBP-3 (Lee et al., 2004).

#### Cellular Uptake of IGFBP-3 Through Caveolae-Mediated Endocytosis

Lipid rafts are microdomains of plasma membranes constituents of which includes sphingolipids (GM1, sphingomyelin, and ceramide), glycolipids, cholesterol, glycophosphatidylinnositol (GPI) anchored proteins (Lajoie and Nabi, 2010). Whereas, caveolae are sub-domains of lipid rafts that are enriched with caveolin proteins. Caveolae can be flask-shaped or inverted flask-shaped structures of 50–100 nm diameters that are of various shapes including flat, vesicular, tubular. Unlike lipid rafts, which are planar and cannot be easily distinguished from the plasma membranes, caveolae are known to associate with a unique family of proteins namely caveolins that can oligomerize to form larger macromolecular complexes and can be visualized under the microscope.

Inhibitors of endocytosis, including lysomotropic agents like chloroquine and monensin (Goltz et al., 1992) that inhibit lysosomal enzymes or microtubule disrupting agents like cholchicine and nocodazole (Jin and Snider, 1993), have been classically used to demonstrate the mechanism of IGFBP-3 internalization into the cells. Cells treated with inhibitors of endocytosis and receptor recycling failed to prevent the nuclear uptake of IGFBP-3, indicating that the cellular uptake of IGFBP-3 was through alternate mechanisms of internalization. A study led by Baxter used breast cancer, T47D cells with Cy3-labeled transferrin and studied the effect on IGFBP-3 localisation in the presence of nocodazole and cholchicine, inhibitors of microtubule assembly, and chloroquine and monensin, lysomotrophic agents (Schedlich et al., 1998). Neither of the types of drugs could prevent the accumulation IGFBP-3 in the nucleus, which indicates an alternate route of IGFBP-3 uptake exists that do not involve receptor-mediated uptake (Schedlich et al., 1998). Baxter proposed the existence of alternate mechanisms of IGFBP-3 internalization, including, cellular uptake through caveolae or direct transport of IGFBP-3 (Schedlich et al., 1998). The uptake of molecules through caveolae could involve either vesicular import or non-vesicular import (Cleves, 1997).

In order to determine if caveolae have a role to play in the internalization of IGFBP-3, inhibitors of caveolae, nystatin and methyl β-cyclodextrin were used. Nystatin and methyl β-cyclodextrin are sterol binding agents that bind to cholesterol, one of the important constituents of caveolae. Inhibitors of caveolae, however, do not impact clathrin-mediated endocytosis, they deplete cholesterol from the membranes. Research led by Cohen, demonstrated that nystatin and methyl β-cyclodextrin reduced the nuclear localisation of IGFBP-3 by ∼40% and cytoplasmic localisation of IGFBP-3 by ∼50% (Lee et al., 2004). The same study also demonstrated that the blocking the of entry of IGFBP-3 through both the mechanisms resulted in the inhibition of endogenous as well as exogenous IGFBP-3 from nuclear localization. When transferrin receptor antibody was used along with methyl β-cyclodextrin, exogenous IGFBP-3 as well as endogenous IGFBP-3 failed to translocate into the nucleus.

Insulin-like growth factor binding protein-3 is not a unique molecule to be internalized through both the classical clatherin-mediated endocytosis as well as through non-classical routes, there are several proteins that have been reported to internalize through both the mechanisms, including influenza virus (Sieczkarski and Whittaker, 2002), cholera toxin (Lee et al., 2004), shiga toxin (Sandvig et al., 1989), epidermal growth factor (EGF) receptor (EGFR) (Mineo et al., 1999), interleukin-2 (Lamaze et al., 2001), insulin receptor (Fagerholm et al., 2009) and GLUT4 (Shigematsu et al., 2003).

#### Cellular Uptake of IGFBP-3 Through Fluid-Phase Endocytosis

Fluid-phase endocytosis can be referred to as pinocytosis or cell drinking. It is the process of non-specific internalization of molecules along with the solvent. Research conducted by Micutkova et al. (2012) has demonstrated that cellular uptake of IGFBP-3 is through all the three major endocytic pathways including, clathrin-coated pits, caveolin-dependent pathway as well as fluid-phase endocytosis. In their study Micutkova examined the effects of siRNA mediated knockdown of clathrin heavy chain for clathrin-coated pits or caveolin-1 for caveolae mediated endocytosis or PAK1 indicative of fluid-phase endocytosis. The study concluded that fluid-phase endocytosis of IGFBP-3 was required for initial time points (20 min). Knocking down either clathrin or caveolin-1 resulted in decrease in the endocytosis of IGFBP-3, which was suggestive of the clathrin-mediated and caveolae-mediated internalization of IGFBP-3. Moreover, the study also demonstrated the requirement of dynamin, a common hub for the endocytic pathways, which was an absolute requirement.

## Igfbp-3 Association With the Proteins of Plasma Membrane

### IGFBP-3 Interacts With Transforming Growth Factor β Receptors (TGF-βR)

Earlier studies have indicated that IGFBP-3 interacts with transforming growth factor-β (TGF-β) signaling cascade (Baxter, 2001; Fanayan et al., 2002). The TGF-β signaling includes the binding of TGF-β with type II TGF-beta receptor (TGF-βRII), which further recruits the type I TGF-β receptor (TGF-βRI). Following the heterodimerization, TGF-βRII phosphorylates and activates TGF-βRI, which can phosphorylate Smad 2 and 3. Phosphorylated Smad 2 and 3 can complex with Smad 4 and cause its translocation to the nucleus to regulate the transcription of genes. Studies utilized T47D breast cancer cells, where TGF-β cannot exert its effects due to the absence of TGF-βRII as a result TGF-β signaling is non-functional. The overexpression of IGFBP-3 results in arresting the transition of cells in G1 phase to S phase, thereby resulting in reduced cell proliferation. However, when exogenous IGFBP-3 is supplemented to T47D cells there was no growth inhibition observed. Expression of TGF-βRII could restore growth inhibition (Fanayan et al., 2002). Using ligand blot analyses, IGFBP-3 has been demonstrated to bind with TGF-βRI, TGF-βRII and type V TGF-β (TGF-βRV) (Kuemmerle et al., 2004) *vide supra*, Figure 2.

### IGFBP-3 Interacts With TGF-βRV

Insulin-like growth factor binding protein-3 has been demonstrated to interact with a cell surface protein, TGF-β receptor V (TGF-βV) also known as IGFBP-3 receptor (IGFBP-3R), which is a Ser/Thr kinase (Leal et al., 1997; Baxter, 2001). 400 kDa IGFBP-3 125I-IGFBP-3 labeled putative receptor could be purified by antiserum against TGF- βV, additionally IGFBP-3 along with 125I-IGFBP-3 could induce growth inhibition in cells thus strongly suggesting that TGF- βV is the putative IGFBP-3 receptor (Leal et al., 1997). Structural studies of purified TGF-βRV demonstrates that it is structurally identical to the low-density lipoprotein receptor related protein-1/activated α_2_M receptor, which is also known as the endocytic receptor (Huang et al., 2003). Although, TGF-βRV is not a classic receptor for IGFBP3, it has been demonstrated to mediate the effects of IGFBP-3 and TGF-β1 ligand. IGFBP-3 serves as a ligand to TGF-βRV leading to growth inhibition and apoptosis (Leal et al., 1997).

### IGFBP-3 Interacts With IGFBP-3 Receptor

Insulin-like growth factor binding protein-3 receptor interacts with IGFBP-3 was discovered using yeast-two hybridization and was cloned by Ingermann et al. (2010). IGFBP-3R is a single spanning membrane receptor that can bind with IGFBP-3 (Ingermann et al., 2010). IGFBP-3 transcript was found to be widely distributed in varying levels in various human tissues. IGFBP-3R could induce apoptosis as well as had antitumor affects *in vivo* (Ingermann et al., 2010). IGFBP-3R has been demonstrated to induce caspase 8-dependent apoptosis in several cancer cell lines by physically interacting with it. Knocking down of/IGFBP-3R could reverse the IGFBP-3/IGFBP-3R induced apoptosis (Ingermann et al., 2010). Ingerman et al. proposed that IGFBP-3R is a novel death receptor involved in mediating caspase-8 apoptotic pathways as well as tumor suppression in breast cancer cells.

It remains to be determined if the internalization of IGFBP-3 through TGF-βV or IGFBP-3R requires clathrin-coated pits.

### IGFBP-3 Binds With Other Unknown Proteins on the Membrane

A study led by Murphy, wherein crosslinking non-glycosylated, biotinylated IGFBP-3 with disuccinimidyl suberate in T47D cells demonstrated that IGFBP-3 could associate with several unknown proteins on the surface of the plasma membrane (Ferry et al., 1999). This study also identified the phosphorylation of IGFBP-3 at the plasma membrane and that both processes could be inhibited by IGF-1. The association of transglutaminase, which possess intrinsic tyrosine kinase activity has been demonstrated to lead to phosphorylation of IGFBP-3 in breast cancer cells (Ferry et al., 1999).

## The Role of Igfbp-3 in the Nucleus and Its Nuclear Binding Partners

### Requirements for the Translocation of Proteins Into the Nucleus

The transport of molecules through the nuclear membrane occurs via the nuclear pore complex (NPC). Molecules lower than the 30 kDa can passively diffuse through NPC in the proper 3D-folded conformation; however, for other proteins the movement is aided by the presence of appropriate signals (Lange et al., 2007; Kim et al., 2017). Firstly, the cargo protein to be transported must be distinguished from the remainder of the proteins through the presence of a conserved stretch of either basic amino acid residues, including lysine, arginine or glutamine known as the classical nuclear localisation sequence (NLS) or proline-tyrosine (PY) stretch which constitute the non-classical NLS (Kim et al., 2017). Similarly, the nuclear proteins that are exported into the cytoplasm possess a consensus sequence of amino acids that are rich in leucine and is referred to as the nuclear export sequence (NES). These proteins can move through the NPC actively, to and fro, through distinct nuclear transport pathways (Robinson-Rechavi et al., 2003; Freitas and Cunha, 2009). The transporters of cargo proteins are known to bind with cargo proteins are collectively known as the karyopherins, the transport factors involved in the import of cargo are generally referred to as importins and the ones involved in export are referred to as exportins. The transporter proteins are part of a huge family of proteins known as importin-β, collectively known as the β-karyopherins.

There are several distinct pathways that are involved in the import of proteins into the nucleus (Schedlich et al., 2000; Freitas and Cunha, 2009; Nardozzi et al., 2010). One of these include the classical NLS-dependent pathway, wherein the β-karyopherins or importin-β interacts with the classical NLS present on the cargo protein and attaches to the karyopherin-α or importin-β, an import adaptor protein. The import complex is eventually internalized into the nucleus through the NPC. Importin-β of the complex interacts with the nucleoporins. Ran, a Ras family of GTPase and a G-protein that can associate with either GDP (inactive state) or GTP (active state) is responsible for energy supply during nuclear import. The active/inactive states are maintained through the G-protein cycle. The binding of either GDP or GTP to Ran is determined by the regulatory proteins namely Ran guanine nucleotide exchange factor (RanGEF) in the nucleus and RAN GTPase activating factor that resides in the cytoplasm (RanGAP). These regulatory proteins functions as molecular switches and ensure the asymmetric distribution of Ran in active/inactive states, wherein RanGTP is enriched in the nucleus and RanGDP is enriched in the cytoplasm, imparting directionality to the cargo import.

Another pathway involves importin-β without involving the adaptor protein, importin-α. Here the cargo possesses a non-classical NLS and can directly associate with importin-β which can interact with nucleoporins and in a Ran-dependent manner dissociate within the nucleus, thus transporting the protein cargo into the nucleus.

Known functions of IGFBP-3 within the nucleus include regulation of gene transcription either directly or through the interaction with nuclear hormone receptors, thereby leading to apoptosis and regulation of DNA repair mechanisms, nonhomologous end joining (NHEJ) double stranded DNA repair.

#### Nuclear Import of IGFBP-3 Through Importin-β

**FIGURE 3:**
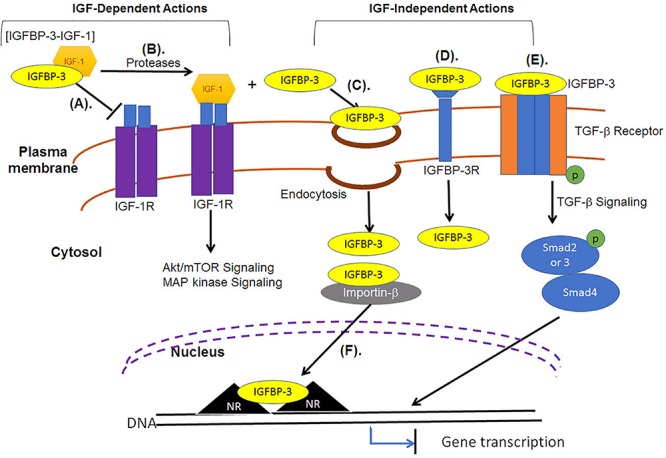
IGF-dependent and IGF-independent roles of IGFBP-3. **(A)** IGF-dependent roles of IGFBP-3 include the transport of IGFs in a binary complex, the state in which it cannot associate with IGF-1R. **(B)** Regulated proteolysis of the binary complex releases IGF, and free IGF can associate with the receptors (IGF-1R) to activate Akt/mTOR or MAP kinase signaling pathways. **(C)** IGFBP-3 can be internalized independent of IGFs into the cytosol through various endocytic mechanisms, including fluid-phase uptake, caveolae-mediated and clathrin-mediated endocytosis. **(D)** IGFBP-3 can associate with membrane proteins, including TGFβVR or IGFBP-3R and induce apoptosis. **(E)** IGFBP-3 can directly associate with TGF-β receptor to regulate the downstream Smad signaling. **(F)** IGFBP-3 can interact with importin-β through its nuclear localization signal (NLS) sequence and is transported into the nucleus, where IGFBP-3 can bind with nuclear receptors (NR) to inhibit gene transcription that can induce apoptosis.

For extracellular IGFBP-3 to localize in the nucleus it must cross the plasma membrane and enter the cytosol. Upon the crossing of plasma membrane, IGFBP-3 has been reported to translocate into the nucleus, by interacting directly with importin-β (Figure 3), without the requirement of adaptor protein, importin-α (Schedlich et al., 2000). Import of full length IGFBP-3 occurred in detergent permeablized plasma membrane but with intact nuclear envelope. Studies led by Baxter demonstrated that antibody specific to importin-β in the presence or absence of cytosol could inhibit the nuclear translocation of IGFBP-3, strongly indicating that importin-β and not importin-α is a minimum requirement during the nuclear import of IGFBP-3. This was in conjunction with the finding that the addition of an analog of GTP that cannot be hydrolyzed (GTP-γS), the nuclear translocation of IGFBP-3 was significantly reduced. The same study also reported that the nuclear import of IGFBP-3 was not observed under conditions lacking ATP generating system. Importin-β recognized NLS in wild type cells, however, cells with mutant NLS failed to recognize mutant form of IGFBP-3. These findings strongly support that NLS, importin-β, ATP and GTP are essential for the nuclear translocation of IGFBP-3. The study failed to provide clear evidence of the involvement of Ran in the nuclear transport of IGFBP-3.

### IGFBP-3 and Nuclear Receptors (NR) Functions as Transcription Factors

Insulin-like growth factor binding protein-3 functions as a transcription factor regulating nuclear hormone receptor activity. NRs are class of proteins, which upon binding with their ligand can interact with specific DNA sequences that can lead to turning ON/OFF of gene expression (Cleves, 1997). NR can be referred to as the ligand-inducible transcription factors. For a review on NR refer to (Mazaira et al., 2018).

The turning ON/OFF of downstream genes can in turn regulate different processes such as development, differentiation, metabolism, cell proliferation, and cell survival. The DNA sequences where the nuclear hormone receptors and their ligand complexes bind are referred to as the hormone response elements (HRE) (Mazaira et al., 2018).

#### IGFBP-3 Interaction With NR, RXR-α and RAR-α

Insulin-like growth factor binding protein-3 has been demonstrated to interact with RXR and RAR-α, thus modulating the RAR signaling in the nucleus. It has been demonstrated that the *N*-terminal and the *C*-terminal domains of IGFBP-3 are involved in this interactions (Schedlich et al., 2007). Liu et al. (2000) have demonstrated that within the nucleus, IGFBP-3 interacts with the nuclear receptors-retinoid X receptor-α (RXR-α). RXR can function both as a homodimer as well as a heterodimer. As a component of a heterodimer complex, RXR can bind with several types of other nuclear hormone receptors including, RAR, vitamin D receptors (VDR), PPAR-**Υ**, liver X receptors, and thyroid hormone receptors. In the study conducted by Liu et al. the role of IGFBP-3 as a regulator of transcription was demonstrated, where the activation was through RXR response elements and the inhibition though RAR response element (Pratt and Pollak, 1993; Ikezoe et al., 2004). Hs578T and MB231 breast cancer cells with basal subtype exhibited a high expression of IGFBP-3, and were unresponsive to all-trans-retinoic acid, a ligand for RAR-α that could be modulated by altering IGFBP-3 levels (Schedlich et al., 2004). In the presence of IGFBP-3 antibody growth inhibition by all-trans-retinoic acid was observed in both the cell lines (Schedlich et al., 2004). This study also reported that the transactivation of RAR response element could be inhibited by IGFBP-3 through the inhibition of RXR-α-RAR-α heterodimer, while the ability of all trans retinoic acid to bind with the complex was maintained, thus supporting the role of IGFBP-3 as a growth stimulator in the breast cancer cells.

#### IGFBP-3 Interactions With Nur77 Activating Intrinsic Apoptosis

In prostate cancer cells exogenous supplementation of IGFBP-3 resulted in induction of apoptosis through the export of orphan nuclear receptors, Nur77 and its binding partner, RXR-α (Agostini-Dreyer et al., 2015). These are the nuclear binding partners of IGFBP-3 that induced the activation of intrinsic apoptotic pathway and released cytochrome c from the mitochondria. This intrinsic apoptosis was mediated though the interactions between IGFBP-3 and Nur77 in the cytoplasmic fraction, and involved the association with RXR-α as it was not observed in RXR-α deficient cells (Lee et al., 2007). IGFBP-3 augmented the association between RXR-α and Nur77 and the translocation of Nur77 from the nucleus into the mitochondria (Lee et al., 2005). IGFBP-3 and Nur77 demonstrated an additive increase in the activation of caspases 3 and 7 leading to increased apoptosis (Lee et al., 2005, 2007). siRNA specific knockdown of Nur77 led to decrease in ansiomycin-induced intrinsic apoptosis by reducing caspase 3 and 7 and their downstream target PARP. In fact, endogenous IGFBP-3 was found to interact directly with RxR-α but not Nur77 under basal conditions, however, the interaction of IGFBP-3 with phosphorylated forms of Nur77 and RXR-α was observed in ansiomycin treated cells. This study supported the role of ansiomycin induced apoptosis in MAC T cells though phosphorylated Nur77 interactions with nuclear IGFBP-3 resulting in its export from the nucleus and the induction of apoptosis (Agostini-Dreyer et al., 2015).

#### IGFBP-3 Binding With Vitamin D Receptor (VDR)

Vitamin D has been reported to increase IGFBP-3 expression in prostate cancer LNCaP cells, an androgen dependent cell line resulting in decreased cell proliferation, in part (Boyle et al., 2001; Krishnan et al., 2003; Peng et al., 2004). The anti-proliferative action of vitamin D occurs through the nuclear receptor vitamin D receptor (VDR). VDR interacts with RXR-α and the heterodimer is recruited on the IGFBP-3 promoter sequence. IGFBP-3 has been reported to reduce the VDR-RXR-dependent transcriptional activity (Krishnan et al., 2003). A hormonally active form of vitamin D, calcitriol is capable of inducing IGFBP-3 expression through the stimulation of p21 and p27 pathways resulting in a decline in cell proliferation (Boyle et al., 2001). IGFBP-3 gene has two vitamin D response elements one at 400 and another at 3350 from the transcription start site (Malinen et al., 2011). Upon the exposure of sense oligodeoxynucleotides along with vitamin D, as expected there was a decline in the cell proliferation and when the cells were incubated with antisense oligonucleotides along with vitamin D, the response was abrogated.

#### IGFBP-3 Interactions With PPAR-Υ

Insulin-like growth factor binding protein-3 has also been demonstrated to directly interact with PPAR-Υ (Moreno-Santos et al., 2017). IGFBP-3 interfered with formation of RXR-α-PPAR-Υ heterodimers and reduced the thiazolidinedione, PPAR-Υ agonist, rosiglitazone stimulated transcriptional activity of PPAR-Υ in adipocytes, 3T3 cells (Chan et al., 2009). In breast cancer cells, IGFBP-3 interactions with PPAR-**Υ** has been demonstrated that results in inhibition of cell growth (Pon et al., 2015). Inhibition of growth by IGFBP-3 and PPAR-**Υ** was higher than either alone in MCF-7, MDA-MB-231, MDA-MB-468 breast cancer cells. The physical interactions between the two proteins occur via the *N*-terminal and *C*-terminal domains of IGFBP-3. Mutations of IGFBP-3 that reduced the binding with PPAR-**Υ** failed to reduce cell growth, similar results were obtained upon silencing transiently using IGFBP-3 specific siRNA and supplementation of exogenous IGFBP-3 restored the cell growth inhibition.

#### IGFBP-3 Interacts With Thyroid Hormone Receptor α1

Using glutathione S-transferase pull down, co-immunoprecipitation and colocalization experiments, IGFBP-3 interactions with thyroid hormone receptor α1 was demonstrated (Jia et al., 2011). This interaction was reported to inhibit the triiodothyronine (T3) responsive gene transcription.

### IGFBP-3 Binding With RNA Pol II Binding Subunit 3 (Rpb3) as a Transcription Factor

Screening the L6 myoblast cDNA expression library using a yeast two-hybrid system was carried out to determine potential interacting partners for IGFBP-3 (Oufattole et al., 2006). Two different IGFBP-3 deletion mutants with 231 residues possessing L61-K291 aminoacids and 111 residues possessing K181-K291 aminoacids were used as bait. One of the novel partners identified amongst several was Rpb3. Gene transcription requires the binding of RNA polymerase II core enzyme complex and Rpb3, one of the 12 subunits is a part of RNA polymerase II. Rpb3 is the third largest subunit and is present away from the core DNA binding domain of the RNA polymerase. Rpb3 is required to recruit specific transcription factors that would initiate the process of transcription. Interaction of the IGFBP-3 mutants with Rpb3 was confirmed using yeast two-hybrid assay by co-transfecting yeast with full length Rpb3, IGFBP-3 deletion mutants and negative controls. The interaction of IGFBP-3 with Rpb3 was further confirmed by co-immunoprecipitation studies. The binding of Rpb3 requires MBD and NLS region of IGFBP-3 protein (Oufattole et al., 2006). Rpb3 binding with IGFBP-3 helps in recruiting transcription factors for the transactivation of genes is suggestive of IGFBP-3’s role in gene transcription.

### IGFBP-3 Interacts With EGFR and DNA-Dependent Protein Kinase (DNA-PK) and the Role of IGFBP-3 in DNA Double Stranded Break Repair-Nonhomologous End Joining (NHEJ)

A study led by Baxter demonstrated that IGFBP-3 could be phosphorylated (Schedlich et al., 2003), later another study led by Cohen demonstrated that the phosphorylation of IGFBP-3 occurs at Ser 156 and is catalyzed by DNA-PK, which is essential for the inhibition of growth and promotion of apoptosis (Schedlich et al., 2003). Phosphorylated IGFBP-3 is required for the nuclear localization and for its interaction with the components within the nucleus. Phosphorylation of IGFBP-3 leads to the loss of affinity for IGF-1 and ensures its release from the IGF-1-IGFBP-3 complex prior to the nuclear localisation of phosphorylated IGFBP-3 (Schedlich et al., 2003). There was no difference between the binding of importin-β and phosphorylated or dephosphorylated forms of IGFBP-3 (Schedlich et al., 2003). The Ser156 phosphorylation of IGFBP-3 not only causes the translocation of IGFBP-3 into the nucleus but also is essential for its interaction with RXR-α within the nucleus.

Epidermal growth factor receptor is RTK that is known to play an important role in the resistance to DNA-damaging radiation therapy and cytotoxic chemotherapy during cancer treatments. DNA protein kinase (DNA-PK) is an essential player during the double stranded DNA repair. DNA-PK is autophosphorylated at Ser2056, which is required for the double stranded DNA repair during non-homologous end joining (NHEJ). Radiation causes the translocation of EGFR into the nucleus, where it interacts with DNA-PK and functions as a transcription factor leading to increased gene transcription of proteins required for double stranded DNA repair during NHEJ, which is essential for reversal of the radiation-induced DNA damage. The radiation-induced nuclear translocation of EGFR and its interaction with DNA-PK, causes phosphorylation of IGFBP-3 at Ser156. Studies by Lin et al. demonstrated that the treatment of etoposide, a DNA damaging drug leads to increased autophosphorylation of DNA-PK at S2956 in the nucleus (Lin et al., 2014). Through direct interaction between IGFBP-3 and EGFR, the complex could be localized into the nucleus, since both possess NLS. Studies using MCF-10A breast cancer cells demonstrated that IGFBP-3 leads to activation of EGFR, which could be internalized within the cells though caveolae-mediated endocytosis (Martin et al., 2003). Additionally, Lin et al. (2014) also demonstrated that etoposide increased the interaction between IGFBP-3, EGFR and DNA-PK, moreover, EGFR tyrosine kinase inhibitor, gefitinib could reverse the effects. IGFBP-3 could interact with EGFR and DNA-PK, thereby, stabilizing the complex or leading to increased autophosphorylation of EGFR. Downregulation of IGFBP-3 could completely abolish the etoposide induced increased EGFR and DNA-PK complex formation and the nuclear localization (Lin et al., 2014). The role of IGFBP-3 as a regulator of human breast cancer growth and survival is complex.

### IGFBP-3 Binds With Non-POU Domain-Containing Octamer Binding Protein (NONO) and Splicing Factor Proline/Glutamine-Rich (SFPQ)

In triple negative breast cancers (TNBCs) responsiveness to chemotherapeutic drugs is inefficient due to ineffective DNA double strand break repair system, NHEJ. Recent studies have determined that the treatment of etoposide to TNBC cell line, HCC1806 and MDA-MB-468 resulted in increased interaction between IGFBP-3 and a DNA/RNA binding protein namely NONO, and its dimerization partner splicing factor proline/glutamine-rich (SFPQ) (de Silva et al., 2019). NONO binding with IGFBP-3 was demonstrated in cell free systems (de Silva et al., 2019). The binding between IGFBP-3, NONO and SFPQ could be blunted by inhibitors of EGFR, gefitinib or with inhibitor of DNA-PK, NU7026 and by PARP inhibitors, veliparib and olaparib. The above-mentioned inhibitors of EGFR, DNA-PK, and PARP could also reduce the NHEJ activity (de Silva et al., 2019). Long noncoding RNA, LINP1 could also abrogate the interactions between IGFBP-3, NONO, and SFPQ (de Silva et al., 2019).

## Igfbp-3 and Its Igf-Independent Role in Apoptosis or Cell Survival

Apoptosis is an event that tips the balance towards cell death rather than survival and can be summarized as the programmed cell death. Apoptosis is mediated through two-cross linked pathways that involve either the cell surface receptors called the death receptors, activated by ligands or emanate from within the cell involving mitochondria, independent of ligands, commonly referred to as the extrinsic or the intrinsic apoptotic pathways, respectively (Elmore, 2007). IGFBP-3 can function both as a suppressor of cell proliferation through the induction of apoptosis as well as in mediating cell proliferation.

### IGF-Independent Role of IGFBP-3 in Apoptosis

The involvement of IGFBP-3 in the inhibition of cell growth is evident when human IGFBP-3 cDNA is transfected into mammalian cell lines like colon cancer cells (MacDonald et al., 1999), human breast cells (Firth et al., 1998a) and mouse fibroblast cells (Cohen et al., 1993). Apoptosis inducing effect of IGFBP-3 has been demonstrated to be an IGF-dependent as well as an IGF-independent process. Treatment of recombinant IGFBP-3 protein to breast cancer cells could inhibit cell growth (Oh et al., 1993). MCF-7 breast cancer cells treated with exogenous IGFBP-3 showed increased apoptosis and this effect could be reversed by the use of non-IGFBP-binding IGF-1analog but not IGF-1 strongly indicating that IGFBP-3 induced apoptosis by sequestering IGF-1 from the receptor (Nickerson et al., 1997). Studies exhibiting the effects of human IGFBP-3, *in vivo* using transgenic mice show results contradictory to the *in vitro* studies using cell line. Human IGFBP-3 transgene has been expressed in colon, kidney and small intestine (Murphy et al., 1995), In comparison with their wild type littermates the human IGFBP-3 transgenic mice exhibit organomegaly of heart, liver and spleen, however, the weights of kidney and brain remained unaltered (Murphy et al., 1995) strongly indicating that IGFBP-3 has organ specific effect on cell growth and proliferation.

Molecules with anti-proliferative properties have been reported to increase IGFBP-3 production, which include TGF-β2 (Yateman et al., 1993), retinoic acid (Fontana et al., 1991; Sheikh et al., 1993), anti-estrogen drugs like ICI 182, 780, tamoxifen (Pratt and Pollak, 1993, 1994), vitamin D (Moriwake et al., 1992; Martin and Pattison, 2000), ribotoxins (Agostini-Dreyer et al., 2015, 2019), TNF-α (Olney et al., 1995), calcitriol, androgen (Sonnenschein et al., 1989; Peng et al., 2004, 2006; Kojima et al., 2006). Serum and growth factor deprivation is also known to induce *de novo* synthesis of IGFBP-3 transcript as well as protein (Grimberg et al., 2002; Takaoka et al., 2006).

It should be noted that there are concentration-dependent effects of IGFBP-3 on cell proliferation observed in breast cancer cells, additionally, TGF-β has been demonstrated to inhibit or augment IGFBP-3 production in a cell line specific manner (McCaig et al., 2002a). Similar dose-dependent behavior has been observed with androgens, at low concentrations androgen are growth stimulating as they fail to induce IGFBP-3 expression (Kojima et al., 2006), on the contrary, at higher concentrations are anti-proliferative due to the ability to induce IGFBP-3 expression (Sonnenschein et al., 1989; Peng et al., 2006).

There are several accumulating *in vitro* and *in vivo* evidences that support the anti-proliferative activity of IGFBP-3 is mediated through apoptosis in an IGF-independent manner (Valentinis et al., 1995; Zadeh and Binoux, 1997; Agostini-Dreyer et al., 2019). IGF-independent effects of IGFBP-3 on apoptosis induction was demonstrated using IGFR1 negative mouse fibroblast cell line, which were either treated with exogenous IGFBP-3 or transfected with IGFBP-3 cDNA (Rajah et al., 1997). Exogenous IGFBP-3 treatment to Hs578T breast cancer cells lacking IGF-1R was demonstrated to have little effect on the inhibition of growth, however, treatment with an analog of ceramide could dramatically enhance apoptosis in an IGF-independent manner (Perks et al., 1999). Ceramide is a physiological mediator of apoptosis which is induced by radiation, TNF-α and interleukin-β (Gill et al., 1997). Caveolin scaffolding domain not only binds with caveolin but also binds with protein kinase A (PKA) and consequently inactivates it (Levin et al., 2007). IGFBP-3 induces apoptosis in Hs578T breast cancer cells by inhibiting PKA upon binding with caveolin through the caveolin scaffolding domain (Perks et al., 2011). A role of Rho and Rho regulated Ser/Thr kinase (ROCK) was found to be involved in mediating apoptosis. The study led by Holly demonstrated that inhibition of PKA, or ROCK or ceramide synthesis could reverse the apoptotic action of IGFBP-3 (Perks et al., 2011). IGFBP-3 could activate Rho, which led to increased ceramide production resulting in MAP kinase induced apoptosis (Perks et al., 2011). IGFBP3 interacts with ECM and has been demonstrated to promote attachment to fibronectin in Hs578T cells (McCaig et al., 2002b). IGFBP-3 was able to accentuate the ceramide-induced cell death, however, in the presence of fibronectin, it reversed the ceramide-induced cell death instead an increased cell survival was observed (McCaig et al., 2002b).

#### IGFBP-3 Interaction With BAX (Bcl-Associated X Protein)

A study focused on understanding the phenomenon of germ cell apoptosis that occurs during spermatogenesis in males reported of the interaction between IGFBP-3 and BAX proteins using dot blot and co-immunoprecipitation. Together this interaction was seen to activate germ cell apoptosis *via* intrinsic apoptotic pathway involving mitochondria (Jia et al., 2010). IGFBP-3 expression in human breast cancer cells is associated with enhanced BAX and Bad expression concomitant with decreased Bcl-2 and Bcl-XL and it is independent of p53 status (Butt et al., 2000).

#### IGFBP-3 Interacts With Humanin

Using yeast-two hybridisation system, humanin was found to be a binding partner of IGFBP-3 and was cloned in a study led by Cohen (Ikonen et al., 2003). A secretory protein in nature, humanin, is a 24 amino acid peptide involved in cell survival. *In vitro* studies have demonstrated that humanin functions against Alzheimer disease related insults to the cell, moreover, IGFBP-3 has been demonstrated to be increased in Alzheimer’s disease (Rensink et al., 2002). Using dot-blot and pull-down experiments the interaction between humanin and IGFBP-3 was confirmed. Additionally, the study also reported that the C-terminal domain of IGFBP-3 comprising of heparin or GAG binding domain was involved in the binding with humanin (Ikonen et al., 2003). Moreover, the interaction of humanin with IGFBP-3 did not alter the IGF-1 binding ability of IGFBP-3. IGFBP-3 is known to possess apoptotic effects as demonstrated by increased cell death monitored through TUNNEL assay, humanin could block the IGFBP-3-induced apoptosis in gliomablastoma cells. Since, IGFBP-3 is present in the extracellular region, cytoplasmic as well as nuclear region of the cell, the regulation between IGFBP-3 and humanin can exist at multiple levels. Humanin has also been reported to interfere with BAX activation thus protecting against apoptosis (Guo et al., 2003). Humanin intereferes with the mitochondrial release of cytochrome c through preventing the translocation of BAX to mitochondria. The mechanistic regulation and repercussions of IGFBP-3-humanin-BAX yet remain to be established.

#### IGFBP-3 Binds With E7 Oncoprotein

Using yeast 2 hybrid system, E7 protein that is encoded by human papillomavirus type 16 was reported to interact with IGFBP-3 (Mannhardt et al., 2000). The transfection of prostate cancer, PC-3 cells with IGFBP-3 resulted in apoptosis, however, PC-3 cells co-transfected with E7 and IGFBP-3 displayed reduced apoptotic cell death. This suggests that IGFBP-3 mediated apoptosis could be inhibited by E7. Furthermore, E7 and IGFBP-3 was found to colocalize within the cells. The cells overexpressing E7 could degrade IGFBP-3 which could be reversed using a proteasome inhibitor.

#### IGFBP-3 and Ribotoxins

Study led by Cohick, determined that ribotoxins, anisomycin leads to increased IGFBP-3 secretion inducing apoptosis. Knockdown of IGFBP-3 is capable of supressing the anisomycin-induced apoptosis (Agostini-Dreyer et al., 2015). In a yet another study by Cohick, determined that ribotoxins, anisomycin and deoxynevalenol induced the transport of IGFBP-3 into the nucleus by augmenting the interaction of IGFBP-3 with importin-β (Agostini-Dreyer et al., 2019). Inhibiting the nuclear transport of IGFBP-3 with importazole could relieve the ribotoxin-induced nuclear translocation of IGFBP-3. Using inhibitors of clathrin-mediated endocytosis, pitstop2 or inhibitors of IGFBP-3 secretion, brefieldin, IGFBP-3 was still reported to translocate into the nucleus. The study concluded that ribotoxin-induced nuclear localized IGFBP-3 is mediated through importin-β pathway and is not the secreted IGFBP-3. Moreover the secreted and intracellular IGFBP-3 was differentially glycosylated as the IGFBP-3 secreted in the conditioned media migrated as a doublet of 38–45 kDa and the intracellular IGFBP-3 migrated as a doublet of 34–38 kDa molecular weight on SDS–PAGE gels (Agostini-Dreyer et al., 2019).

### Cytoplasmic Export of Nuclear IGFBP-3 and Apoptosis

Insulin-like growth factor binding protein-3 is the only known protein of all the IGFBPs to exhibit translocation from the nucleus into the cytoplasm. Following the secretion, IGFBP-3 is internalized into the cytoplasm and translocated into the nucleus, later detected in the cytoplasm suggestive of its ability to be exported from the nucleus into the cytoplasm. Paharkova-Vatchkova, et al. reported the presence of a putative nuclear export sequence (NES) in IGFBP-3, which corresponds to amino acids 217–228 (Paharkova-Vatchkova and Lee, 2010). This includes leucine-rich sequences that are analogous to HIV Rev and p53. Using mutagenesis experiments, the identification of Leu 197 and Leu 200, part of *C*-terminal domain of IGFBP-3 was found to be critical in the export of nuclear IGFBP-3 into the cytoplasm. In the prostate cancer cells, wild type (wt) IGFBP-3 was equally distributed in the cytoplasmic and nuclear fractions, whereas, mutants of IGFBP-3 at NES, Leu 197 and Leu 200 demonstrated an increased expression of IGFBP-3 in the nuclear fraction in comparison with the wt. These results suggest that NES IGFBP-3 mutant failed to get exported into the cytoplasm from the nucleus. Moreover, NES mutants also demonstrated a decrease in the oligonucleosomal fragmentation, which is indicative of apoptosis, clearly suggesting that the export of IGFBP-3 from the nucleus is required for the induction of apoptosis. Impaired nuclear export of IGFBP-3 caused heterodimers of Nur77 and RXR to be retained in the nucleus, no export to the cytosol was observed and as a result the antiapoptotic activity of IGFBP-3 was abolished. Using cell fractionation studies, IGFBP-3 has been reported to localize in the endoplasmic reticulum and the mitochondria. IGFBP-3 interacted with Nur77 in the ER. A confounding factor of the study include the inability to differentiate the IGFBP-3 exported from the nucleus to ER and the *de novo* ribosomal synthesis of IGFBP-3 on ER (Paharkova-Vatchkova and Lee, 2010). In the endoplasmic reticulum and mitochondria, IGFBP-3 could possibly play an essential role in triggering ER-stress, oncogene expression, death receptor ligation and oxidative stress induced apoptosis (Paharkova-Vatchkova and Lee, 2010).

### IGFBP-3 and Its IGF-Independent Role in Survival

**FIGURE 4:**
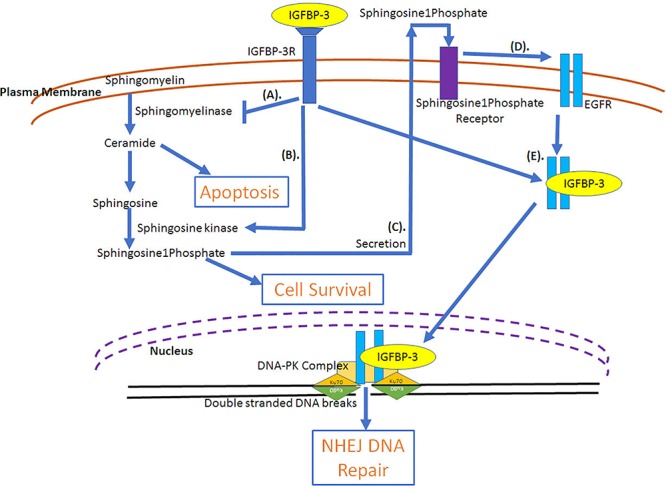
IGF-Independent involvement of IGFBP-3 in cell survival pathways. **(A)** IGFBP-3 potentiates cell survival through the inhibition of ceramide (inducer of apoptosis) synthesis by the inactivation of sphingomyelinase and **(B)** by the activation of sphingosine kinase that results in the formation of sphingosine 1 kinase from sphingosine. While sphingosine can induce apoptosis, sphingosine 1 phosphate can potentiate cell survival. **(C)** Sphingosine 1 phosphate is secreted and can bind with its receptor, which can **(D)** transactivate EGFR, growth survival receptor. **(E)** Due to radiation, through the lipid rafts IGFBP-3 and EGFR are internalized into the cell and translocated into the nucleus, where EGFR-IGFBP-3 can complex with DNA-PK (DNA-protein kinase) facilitating DNA double stranded break repair through non-homologous end joining (NHEJ). DNA-PK complex includes Ku70 and Ku80 that encircles 20 bp DNA and kinase, which is the catalytic subunit, DNA-PKcs.

The role of IGFBP-3 in augmenting cell proliferation and survival is due to its ability to deliver IGFs to its receptors on the cell (IGF-dependent effects), IGF-independent effects on cell growth have also been demonstrated. IGFBP-3 could increase cell growth in breast cancer cells in an IGF-independent manner (Martin et al., 2003; Butt et al., 2004). In MCF-10A cells, IGFBP-3 induced growth through the activation of EGFR receptor phosphorylation/activation *via* MAP kinase signaling and activation of p44/42 signaling (Martin et al., 2003). Similar effect was observed in T47D, breast cancer cells stably transfected with IGFBP-3, where long-term cultures could induce cell growth, however, short-term cultures failed to induce growth (Butt et al., 2004). The increased growth was due to increased stimulation of EGFR signaling. Additionally, increased passage number of stably transfected IGFBP-3 in T47D cells showed increased growth, which indicates that breast cancers can alter their response to IGFBP-3 at different tumorigenicity (Butt et al., 2004). The evidence to this is clear as IGFBP-3 has been demonstrated to be present in high levels in several cancers, including breast (Valentinis et al., 1995; Rocha et al., 1997; Sheen-Chen et al., 2009), squamous cell lung (Kettunen et al., 2004), clear cell renal (Chuang et al., 2008) and pancreatic (Xue et al., 2008) cancers. As a matter of fact, in breast cancer there are reports indicating high expression of IGFBP-3 with poor prognosis and survival outcome (Valentinis et al., 1995; Rocha et al., 1997; Sheen-Chen et al., 2009). On the contrary, in head and neck cancer (Papadimitrakopoulou et al., 2006) and hepatocellular carcinoma (Aishima et al., 2006) low IGFBP-3 expression has been associated with shorter disease-specific and disease-free survival outcome. One of the mechanisms explaining the ability of IGFBP-3 to potentiate breast cancer cell growth is the ability of IGFBP-3 to activate sphingosine kinase, an enzyme that incorporates phosphate group into sphingosine and the inhibition of sphingomyelinase, an enzyme that generates ceramide from sphingosine (Figure 4). Ceramide has been demonstrated to cause cell cycle arrest and apoptosis (Perks et al., 2002). Sphingosine is a growth inhibitor, however, sphingosine1phosphate acts as a growth stimulator (Granata et al., 2004). IGFBP-3 resulted in the increased doxorubicin-induced apoptosis due to increased ceramide levels in contrast to increased cell survival under serum deprived conditions due to decreased ceramide levels in HUVEC, endothelial cells (Granata et al., 2004). IGFBP-3 induced survival is mediated through IGF-1R (Granata et al., 2004). Sphingosine1phosphate is secreted following which it interacts with its receptor, Edg-3, that can transactivate EGFR in a metallomatrix protease-dependent manner (Sukocheva et al., 2006). IGFBP-3 inhibited the EGF-induced growth of breast cancer cells when cultured on plastic or laminin but could augment EGF-induced cell proliferation when cultured on fibronectin (McIntosh et al., 2010). In breast epithelial cells expression of IGFBP-3 resulted in the activation of sphingosine kinase, which is the mechanism of transactivation of EGFR. siRNA mediated knockdown of sphingosine kinase1 but not sphingosine kinase2 could prevent the transactivation of EGFR (Sukocheva et al., 2006). It was also observed that silencing of shingosine1phosphate receptor1 and 3 could prevent the transactivation of EGFR but not sphingosine1phosphate receptor 2 (Sukocheva et al., 2006).

Further to the expansive role of IGFBP3 in eliciting both deleterious or protective effect, it plays an important role in ocular cells by conferring vascular protection to sites of injury by augmenting proliferation, migration, and differentiation of vascular progenitor cells. Endothelial progenitor cells displaying the CD34^+^ surface were observed to display increased migratory behavior in a dose dependent manner when IGFBP3 was added exogenously which also resulted in increased endothelial nitric oxide synthase activity (Nguyen et al., 2013). Furthermore, research led by Maria Grant demonstrated that administration of IGFBP3 displayed a dose dependent reduction in artery constriction placed under intraluminal pressure. The release of NO was found to be independent of calcium mediated NO release (Nguyen et al., 2013).

#### IGFBP-3 Interacts With GRP78 and Induces Autophagy

Using yeast-two hybrid system it was determined that IGFBP-3 interacts with glucose-regulated protein 78 (GRP78) also known as binding immunoglobulin protein (BiP) (Li et al., 2012). GRP78 or BiP functions in the regulation of unfolded protein response (UPR) due to the accumulation of misfolded proteins within the ER, however, the role of IGFBP-3 was not supported in ER. On the contrary, the role of IGFBP-3 as a binding partner was to modulate apoptosis. The interaction of IGFBP-3 with GRP78 disrupted the GRP78 interaction with caspase-7 thereby activating it for the induction of apoptosis. Either the overexpression of IGFBP-3 or the transient reduction of GRP78 through the use of siRNA resulted in a decline in the cell viability (Li et al., 2012). The interaction between IGFBP-3 and GRP78 was confirmed using co-immunoprecipitation, nanomolar binding assay and glutathione S-transferase (GST) pull-down assay. From the GST pull-down assay, it was established that the ATPase domain of GRP78 or BiP was required to interact with IGFBP-3. In breast cancer cells, IGFBP-3 was found to promote autophagy thereby augmenting cell survival during nutrition deprivation and hypoxia (Grkovic et al., 2013).

## Concluding Remarks

While the earlier discovery of IGFBP-3’s endocrine function in transporting IGFs as a ternary complex in the blood stream bound with ALS, is a major mechanism of IGF-dependent somatic growth. The IGF-independent role of IGFBP-3 involving its association with the plasma membrane and its internalization into the cell and the nucleus, are important in regulating various important cellular functions. The ability of 8-22-mer peptides generated from the *C*-terminal domain of IGFBP-3 can specifically target cancer cells and it can be tagged with unrelated proteins or therapeutic drugs to efficiently transport within the cells, could prove to be an important biotechnological application of IGFBP-3 molecule. The ability of IGFBP-3 CPPs to specifically target cancer cells is a major advantage in comparison with other CPPs.

Recent discoveries have identified IGFBP-3 to play dual function of a gate-keeper (induction of apoptosis and cell cycle arrest) as well as care-taker (DNA repair through interaction with DNA-PK, induction of autophagy by interaction with GRP78 and the ability to regulate sphingolipids required for the cell survival pathways) through mechanisms independent of IGFs. However, the impact of IGFBP-3 has been difficult to assess as there are no diseases-causing mutations of IGFBP-3 that exist. The ability of IGFBP-3 to interact with several other proteins is a dynamic property exhibiting its multifaceted role in the modulating several critical cellular functions that are independent of IGFs and are context-dependent. Nuclear IGFBP-3 can function as a direct as well as indirect transcription factor and gene transcription, additionally, it also has a role to play in the process of DNA repair. The ability of IGFBP-3 to interact with RXR-α homo and heterodimers with other distinct proteins to form nuclear receptors, signifies the relevance of IGFBP-3 as a regulator of gene transcription. IGFBP-3 can function as an activator of gene transcription when interacting with RXR-α, on the contrary IGFBP-3 interaction with RAR or VDR can result in gene transcription inhibition.

The pleiotropic nature of IGFBP-3, whereby, it interacts with plethora of partners is an important property possessed by the molecule that has not only provided new insights into understanding the basic mechanisms of several cellular processes but could also play an essential role in deciphering the unknown complex mechanism(s) of IGFBP-3, thus establishing it as potential targets in several diseases including cancer and other metabolic diseases.

## Author Contributions

SV conceived, designed, and wrote the manuscript. AB, KP and AS have contributed towards writing of the manuscript.

## Conflict of Interest

SV was employed by company VastConInc. The remaining authors declare that the research was conducted in the absence of any commercial or financial relationships that could be construed as a potential conflict of interest.
